# A red blood cell‐derived bionic microrobot capable of hierarchically adapting to five critical stages in systemic drug delivery

**DOI:** 10.1002/EXP.20230105

**Published:** 2023-12-10

**Authors:** Ya‐Xuan Zhu, Hao‐Ran Jia, Yao‐Wen Jiang, Yuxin Guo, Qiu‐Yi Duan, Ke‐Fei Xu, Bai‐Hui Shan, Xiaoyang Liu, Xiaokai Chen, Fu‐Gen Wu

**Affiliations:** ^1^ State Key Laboratory of Digital Medical Engineering Jiangsu Key Laboratory for Biomaterials and Devices School of Biological Science and Medical Engineering Southeast University Nanjing Jiangsu People's Republic of China; ^2^ Shanghai Tenth People's Hospital Shanghai Frontiers Science Center of Nanocatalytic Medicine School of Medicine Tongji University Shanghai People's Republic of China; ^3^ The Cancer Hospital of the University of Chinese Academy of Sciences (Zhejiang Cancer Hospital) Hangzhou Institute of Medicine (HIM) Chinese Academy of Sciences Hangzhou Zhejiang People's Republic of China; ^4^ School of Chemistry Chemical Engineering and Biotechnology Nanyang Technological University Singapore Singapore

**Keywords:** deep tumour penetration, dual‐controllable, laser‐triggered drug release, magnetic guidance, microrobot

## Abstract

The tumour‐targeting efficiency of systemically delivered chemodrugs largely dictates the therapeutic outcome of anticancer treatment. Major challenges lie in the complexity of diverse biological barriers that drug delivery systems must hierarchically overcome to reach their cellular/subcellular targets. Herein, an “all‐in‐one” red blood cell (RBC)‐derived microrobot that can hierarchically adapt to five critical stages during systemic drug delivery, that is, circulation, accumulation, release, extravasation, and penetration, is developed. The microrobots behave like natural RBCs in blood circulation, due to their almost identical surface properties, but can be magnetically manipulated to accumulate at regions of interest such as tumours. Next, the microrobots are “immolated” under laser irradiation to release their therapeutic cargoes and, by generating heat, to enhance drug extravasation through vascular barriers. As a coloaded agent, pirfenidone (PFD) can inhibit the formation of extracellular matrix and increase the penetration depth of chemodrugs in the solid tumour. It is demonstrated that this system effectively suppresses both primary and metastatic tumours in mouse models without evident side effects, and may represent a new class of intelligent biomimicking robots for biomedical applications.

## Graphical abstract

This research develops an “all‐in‐one” red blood cell‐derived microrobot capable of stably circulating in blood vessels, targeting tumour regions under magnetic guidance, releasing therapeutic cargoes upon laser trigger, and facilitating drug penetration by damaging extracellular matrix in tumour tissues. This system addresses critical challenges during systemic drug delivery and represents a new class of intelligent biomimicking robots for biomedical applications.

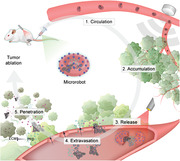

## 1 INTRODUCTION

The progress in the field of precise tumour‐targeted drug delivery has revolutionized our understanding of drug design and profoundly influenced the development trajectory of cancer therapy. In general, drug delivery systems (DDSs) can experience several stages after intravenous administration, including systemic circulation, tumour accumulation, blood vessel extravasation, drug release, and penetration, and their efficiency at each step should be maximized to achieve better therapeutic outcomes.^[^ ^1^, ^2^, ^3^, ^4^, ^5^ ^]^ To meet this goal, a variety of strategies have been designed to optimize the parameters of DDSs from multiple aspects.^[^ ^6^, ^7^, ^8^ ^]^ For example, extending the circulation time of DDSs in blood can be realized either by tuning their size and morphology,^[^ ^9^, ^10^ ^]^ or by modifying them with polymers^[^ ^11^, ^12^ ^]^ such as polyethylene glycol (PEG) or red blood cell (RBC) membrane^[^ ^13^, ^14^ ^]^ to reduce their capture by the mononuclear phagocyte system. Tumour accumulation can be improved by introducing chemical ligands to the DDSs for enhanced tumour recognition or applying an external field (e.g., magnetic field) to direct the distribution of DDSs.^[^ ^15^, ^16^ ^]^ Rational design of DDSs with responsive functional units that can respond to the tumour microenvironment as well as external stimuli will guarantee on‐demand drug release in a spatiotemporally controllable fashion.^[^ ^17^, ^18^, ^19^, ^20^, ^21^ ^]^ Furthermore, inhibiting the formation of extracellular matrix (ECM) in solid tumours or enhancing the drug transportation between cancer cells has been reported to facilitate the deep penetration of drugs.^[^ ^22^, ^23^, ^24^, ^25^ ^]^ Unfortunately, it is still challenging for a single DDS to meet all the requirements during the entire course of drug delivery. Thus, developing smart and controllable DDSs that can responsively adapt to different stages is important for efficiently delivering drugs into the tumour.

Micro/nanorobots have been widely used in diverse biomedical applications such as surgery,^[^ ^26^ ^]^ drug delivery,^[^ ^27^, ^28^, ^29^, ^30^, ^31^, ^32^ ^]^ and biosensing.^[^ ^33^ ^]^ Compared with traditional drug carriers, micro/nanorobots are highly guidable and manoeuvrable in response to different external stimuli,^[^ ^34^, ^35^, ^36^, ^37^, ^38^, ^39^ ^]^ including magnetic field,^[^ ^40^, ^41^ ^]^ laser,^[^ ^42^ ^]^ and ultrasound,^[^ ^43^ ^]^ which can serve as power sources to drive the movement of micro/nanorobots or to trigger the release their payloads. In particular, biomimetic micro/nanorobots based on natural cells (e.g., RBCs,^[^ ^44^ ^]^ platelets,^[^ ^45^, ^46^ ^]^ leukocytes,^[^ ^47^ ^]^ and natural killer cells^[^ ^48^ ^]^) have the potential to inherit the biological features from their source cells to a certain extent and better adapt to the complicated in vivo environment than artificial materials. For example, neutrophil‐based micromotors take advantage of the tropism of neutrophils to migrate toward inflammatory or tumour tissues^[^ ^49^ ^]^ and microphage‐based micromotors can inherit the ability to modulate immune microenvironments.^[^ ^50^ ^]^ As for RBCs, they have been widely exploited for the construction of micro/nanorobots as intelligent DDSs, benefiting from their simple structure, large cavity, and long blood circulation time.^[^ ^51^, ^52^, ^53^ ^]^

**SCHEME 1:**
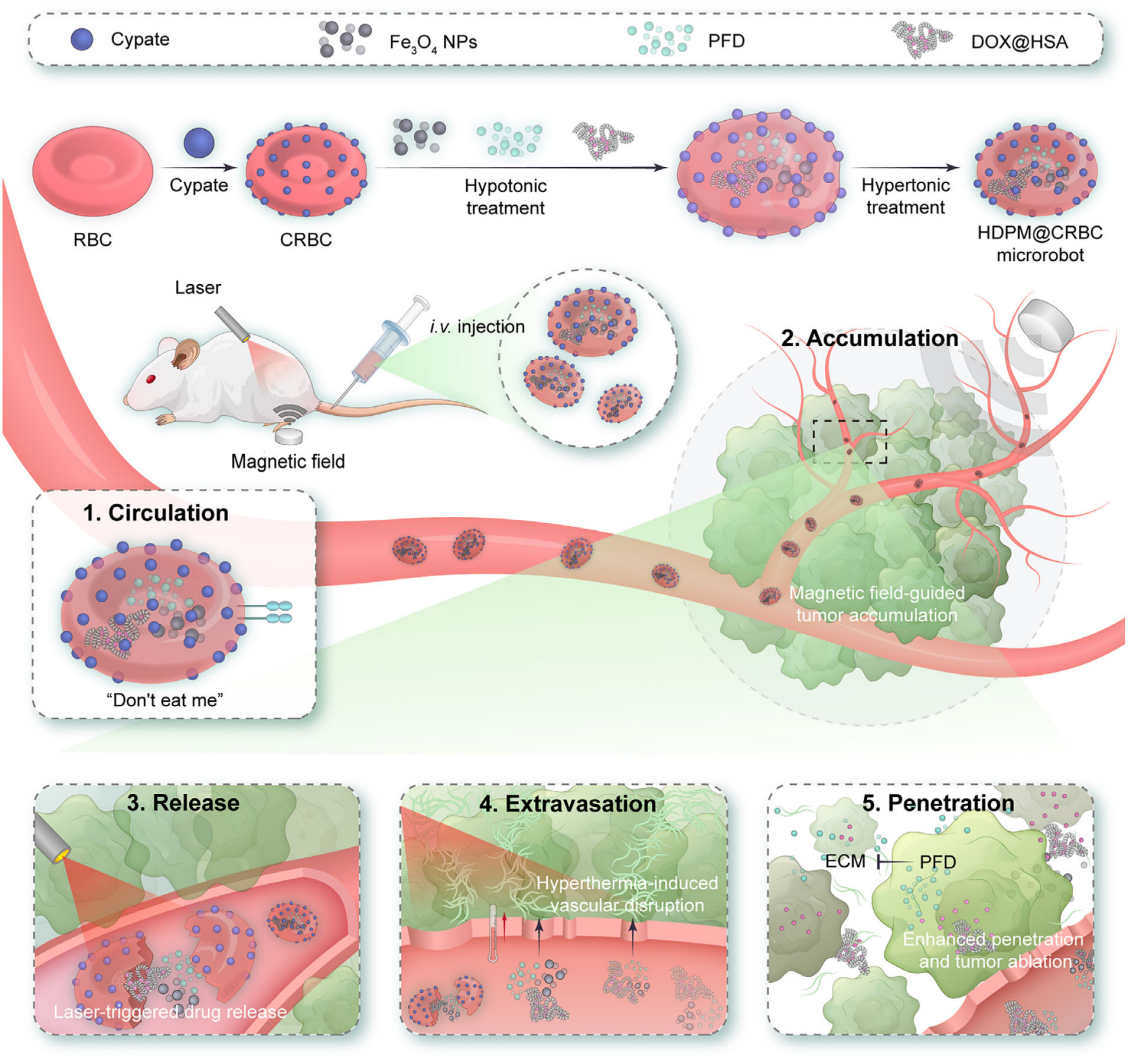
Schematic illustration of the fabrication procedure and the magnet/laser‐controlled anticancer process of the RBC‐based microrobot (HDPM@CRBC).

In this work, we report an RBC‐based light/magnet‐responsive microrobot for precisely controllable and hierarchical drug delivery (Scheme 1). The framework of the as‐designed DDS was fabricated by covalently conjugating cypate, a near‐infrared (NIR) light‐triggerable cyanine dye, onto the surface of RBCs and encapsulating magnetic nanoparticles (NPs) inside their cavities for magnetic control. This dually responsive microrobot was further loaded with the human serum albumin‒doxorubicin complex (denoted as DOX@HSA), a representative chemodrug, and pirfenidone (PFD), an antifibrosis agent capable of regulating the ECM of solid tumours, for synergistic cancer treatment. The as‐prepared DDS (termed HDPM@CRBC) exhibited a long circulation time because of the preserved surface characteristics from natural RBCs. Under the guidance of magnetic field, HDPM@CRBCs could efficiently accumulate at the tumour tissue and release the loaded drugs in response to laser irradiation. The released PFD could inhibit the formation of ECM and facilitate the deep penetration of DOX@HSA for efficient tumour ablation. To sum up, the superb adaptability and flexibility of the microrobot under the control of magnetic field and laser enable it to overcome the multiple biological barriers and realize highly efficient cancer therapy.

## 2 RESULTS AND DISCUSSION

**FIGURE 1:**
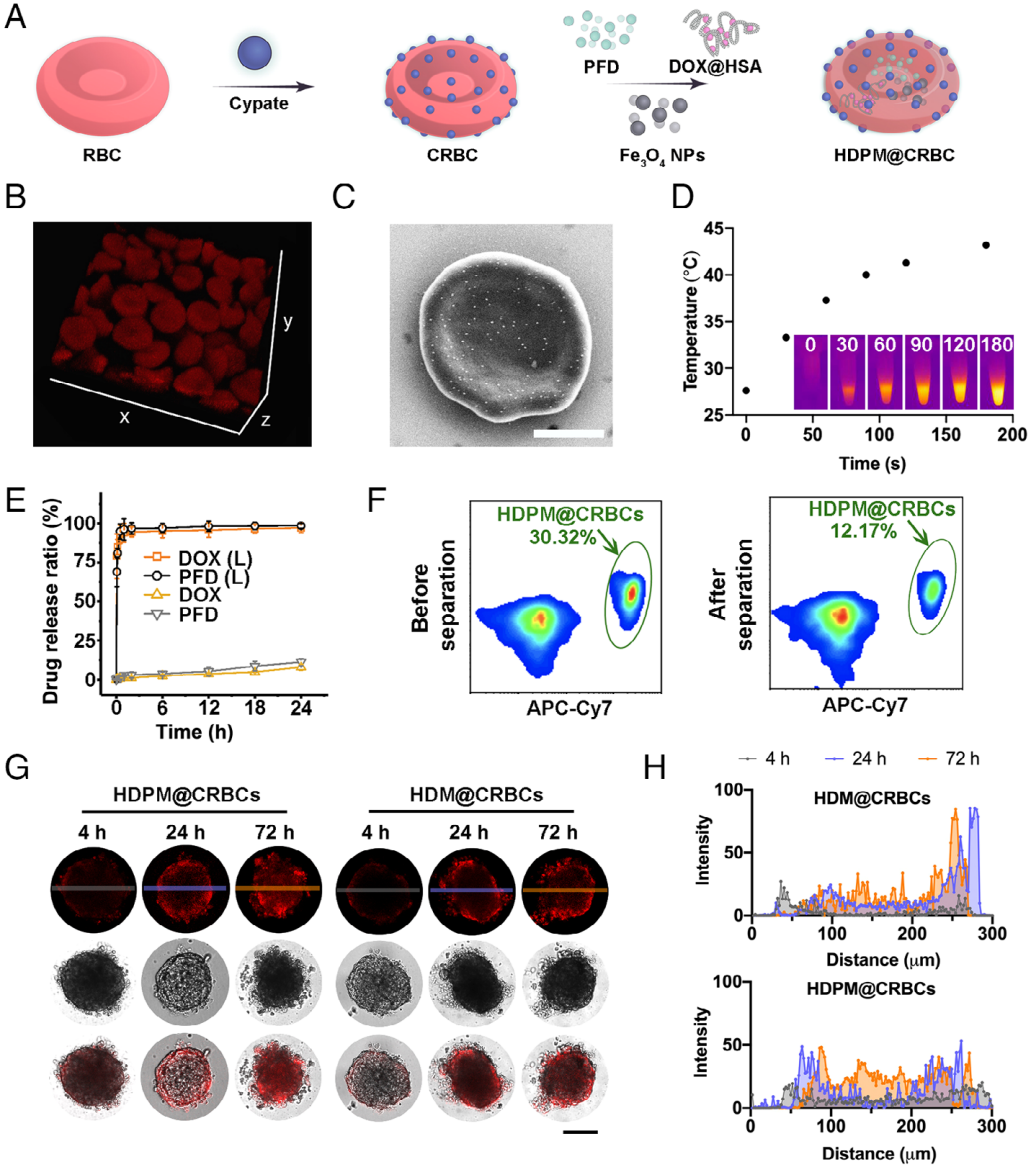
Characterization of HDPM@CRBCs. A, Preparation procedure of the HDPM@CRBC microrobot. B, Representative 3D confocal image of HDPM@CRBCs. C, SEM image of a representative HDPM@CRBC. Scale bar = 2 µm. D, Time‐dependent temperature elevation curve of an HDPM@CRBC suspension under continuous 808 nm laser irradiation (300 mW cm^−^^2^), and infrared thermal images at indicated time points (inset). E, DOX and PFD release profiles of HDPM@CRBCs in the presence or absence of laser irradiation (abbreviated as L in the figure legend). F, Flow cytometric results of the mixture of HDPM@CRBCs (APC‐Cy7‐positive) and RBCs (APC‐Cy7‐negative) before and after magnetic separation. G, Confocal images of MTSs acquired at 4, 24, or 72 h after being treated with the supernatants of laser irradiation‐treated HDPM@CRBCs or HDM@CRBCs. Scale bar = 200 µm. H, Quantitative results of the distribution of DOX fluorescence signals corresponding to G.

To prepare the magnetic field/laser dual‐controllable microrobot, the RBCs were first conjugated with cypate and then loaded with the magnetic nanoparticles and drugs (Fe_3_O_4_ NPs and PFD) (Figure 1A). First, we modified RBCs with cypate to endow the microrobots with laser responsiveness. Cypate is a kind of cyanine dyes and has been widely adopted for achieving diverse biomedical applications, such as bioimaging and photothermal/photodynamic therapy. Cypate contains two carboxyl groups, which are suitable to be activated to react with the abundant amino groups on the RBC surface. Besides, the NIR absorbance of cypate ensures the microrobots to possess light responsiveness under even deep‐seated tissues. To be specific, cypate‐modified RBCs (CRBCs) were prepared by conjugating cypate on the surface of RBCs via covalent conjugation. Briefly, cypate was first mixed with *N*‐hydroxysulfosuccinimide sodium salt (sulfo‐NHS) and 1‐ethyl‐3‐(3‐dimethylaminopropyl)‐carbodiimide hydrochloride (EDC•HCl) to activate the carboxyl groups, and then conjugated to the surface of RBCs via the carboxyl‒amine reaction. To maximize the efficiency of light responsiveness of the obtained CRBCs without distorting their original morphology, we optimized the concentration of cypate for RBC modification. As shown in Figure S1a,b, the amount of conjugated cypate and grafting efficiency were evaluated at various concentrations of cypate. Specifically, as revealed by Figure S1a, the quantity of grafted cypate on the RBC membrane steadily increased, as the cypate concentration increased from 10 to 100 µg mL^−1^, and then reached a plateau at a concentration of 100 µg mL^−1^ or higher, suggesting that the grafting density of cypate was saturated. A similar trend was also revealed by the flow cytometric analysis (Figure S1c). Then, the photothermal efficiency of CRBCs was investigated by measuring the temperature changes of CRBC suspensions during laser irradiation (Figure S1d). To be noticed, we used a mild NIR laser (808 nm, 300 mW cm^−^^2^) in our experiments to avoid the laser‐induced tissue damage. As expected, the CRBCs that were prepared with higher feeding concentrations of cypate exhibited larger temperature increments under continuous laser irradiation (808 nm, 300 mW cm^−^^2^). Next, we evaluated the laser‐triggered haemolysis of CRBCs caused by the photothermal effect. As shown in Figure S1e, surface modification of RBCs with cypate at a feeding concentration of 75 µg mL^−1^ or higher could induce almost complete haemolysis after laser irradiation, demonstrating the efficient laser‐triggered RBC membrane disruption. Based on the above results, 75 µg mL^−1^ cypate was chosen for the construction of CRBCs.

Just like bare RBCs, these CRBCs may not have a desirable tumour‐homing property if they are administered into blood vessels. To address this issue, a magnetic field‐guiding module (Fe_3_O_4_ NPs) was introduced into the inner cavity of CRBCs. Flow cytometric results indicated that the forward scatter (FSC, reflecting particle size) signals of RBCs were not significantly altered after the encapsulation of Fe_3_O_4_ NPs (Figure S2a), while the side scatter (SSC, indicating the complexity of a particle) signals dramatically increased (Figure S2b), which clearly demonstrated the successful loading of Fe_3_O_4_ NPs into the RBCs. Furthermore, as shown in Figure S2c, the magnetic NP‐loaded RBCs (termed M@RBCs) showed elevated magnetic separation efficiencies at higher Fe_3_O_4_ NP loading concentrations, and the optimal loading concentration of Fe_3_O_4_ NPs was determined to be 200 µg mL^−^^1^, to ensure ideal separation efficiency and cause little change to the morphology of RBCs. The confocal images also demonstrated that the M@RBCs (red) were separated from the natural RBCs (yellow) after treated with magnetic field (Figure S2d). No evident change of RBC morphology was observed under this circumstance. Inspired by the above results, we then encapsulated Fe_3_O_4_ NPs into the inner cavity of CRBCs to obtain a laser‐controllable and magnet‐guidable drug delivery system (i.e., M@CRBCs). Then, we monitored the movement of M@CRBCs under magnetic field. The movement trajectories of the M@CRBCs driven by a magnet were shown in Figure S3, which intuitively proved the superb magnetic field responsiveness of M@CRBCs.

Aside from tumour accumulation and drug release, the tumour penetration of drugs is also one of the critical factors that can determine the final tumour ablation outcome. ECM is a major component of solid tumours and actively participates in their growth, immune escape, and metastasis. Tumour ECM with abnormal rigidity can serve as a physical barrier and prevent drugs from penetrating deeply into the tumour. Furthermore, ECM also squeezes blood vessels and causes the decline of blood flow, limiting the tumour uptake of delivered drugs. In this context, inhibiting the formation of ECM is promising to efficiently enhance the tumour accumulation and penetration of drugs and earn better therapeutic outcomes. To achieve this goal, the M@CRBCs were further loaded with a chemotherapeutic agent, DOX@HSA (abbreviated as HD), and an antifibrosis drug, PFD, to afford the final DDS (denoted as HDPM@CRBCs). The synergistic efficacy of PFD and DOX@HSA was first evaluated via multicellular tumour spheroids (MTSs). We confirmed that PFD could inhibit the formation of ECM, because the tumour penetration depth of DOX@HSA was markedly improved in the presence of PFD, especially when the PFD concentration reached 100 µg mL^−1^ or higher (Figures S4 and S5). Moreover, the addition of PFD concomitantly enhanced the oxygen permeation (Figure S6) into the MTS, which further validated the ECM‐inhibiting effect of PFD. As expected, the enhanced tumour penetration of DOX@HSA also resulted in a higher cell killing efficiency against MTSs (Figure S7).

We then compared the morphology of HDPM@CRBCs with that of natural RBCs. 3D confocal imaging and scanning electron microscopy (SEM) unravelled that the morphology of RBCs was not distorted after being fabricated into HDPM@CRBCs (Figure 1B,C). Thus, it is reasonable to extrapolate that the HDPM@CRBCs may share a similar hemodynamic behaviour with their native counterparts.

Next, we investigated whether the HDPM@CRBCs could achieve efficient drug release upon laser irradiation. In our design, cypate is introduced to disrupt the RBC membrane via its photothermal effect and to trigger the release of encapsulated drugs, and thus the heat generated during laser irradiation determines the efficiency of laser‐triggered drug release. It was observed that HDPM@CRBCs showed efficient temperature elevation upon 808 nm laser irradiation (Figure 1D), ensuring the burst release of DOX@HSA and PFD (Figure 1E). In marked contrast, the HDPM@CRBCs showed less than 15% drug release within 24 h under the dark condition. The magnetic responsiveness of HDPM@CRBCs was also evaluated to ensure their preferential accumulation in tumour tissues under magnetic guidance. As shown in Figure 1F, the mixture of HDPM@CRBCs (APC‐Cy7‐positive) and natural RBCs (APC‐Cy7‐negative) was first subjected to a magnetic field to separate the HDPM@CRBCs from natural RBCs. The mixture was analyzed via flow cytometry before and after magnetic separation to quantify the separation efficiency. After being treated with magnetic field, the rate of HDPM@CRBCs was significantly lowered from 30.3% to 12.2%, indicating that HDPM@CRBCs could be efficiently separated from the mixture. This result demonstrated the efficient magnetic field responsiveness of HDPM@CRBCs, and ensured their capability of in vivo magnetic field‐guided tumour targeting. Furthermore, we sought to investigate whether the PFD, after being released from HDPM@CRBCs, could enhance the penetration depth of DOX@HSA in MTSs. To this end, the HDPM@CRBC suspension was subjected to laser irradiation and the supernatant was collected to treat MTSs. Confocal images and the corresponding quantitative results revealed that DOX@HSA penetrated into the core region of MTS in the HDPM@CRBCs group (Figure 1G,H), while DOX@HSA in the HDM@CRBCs group (lacking PFD) was largely restricted in the peripheral region, demonstrating the critical role of PFD coencapsulation.

**FIGURE 2:**
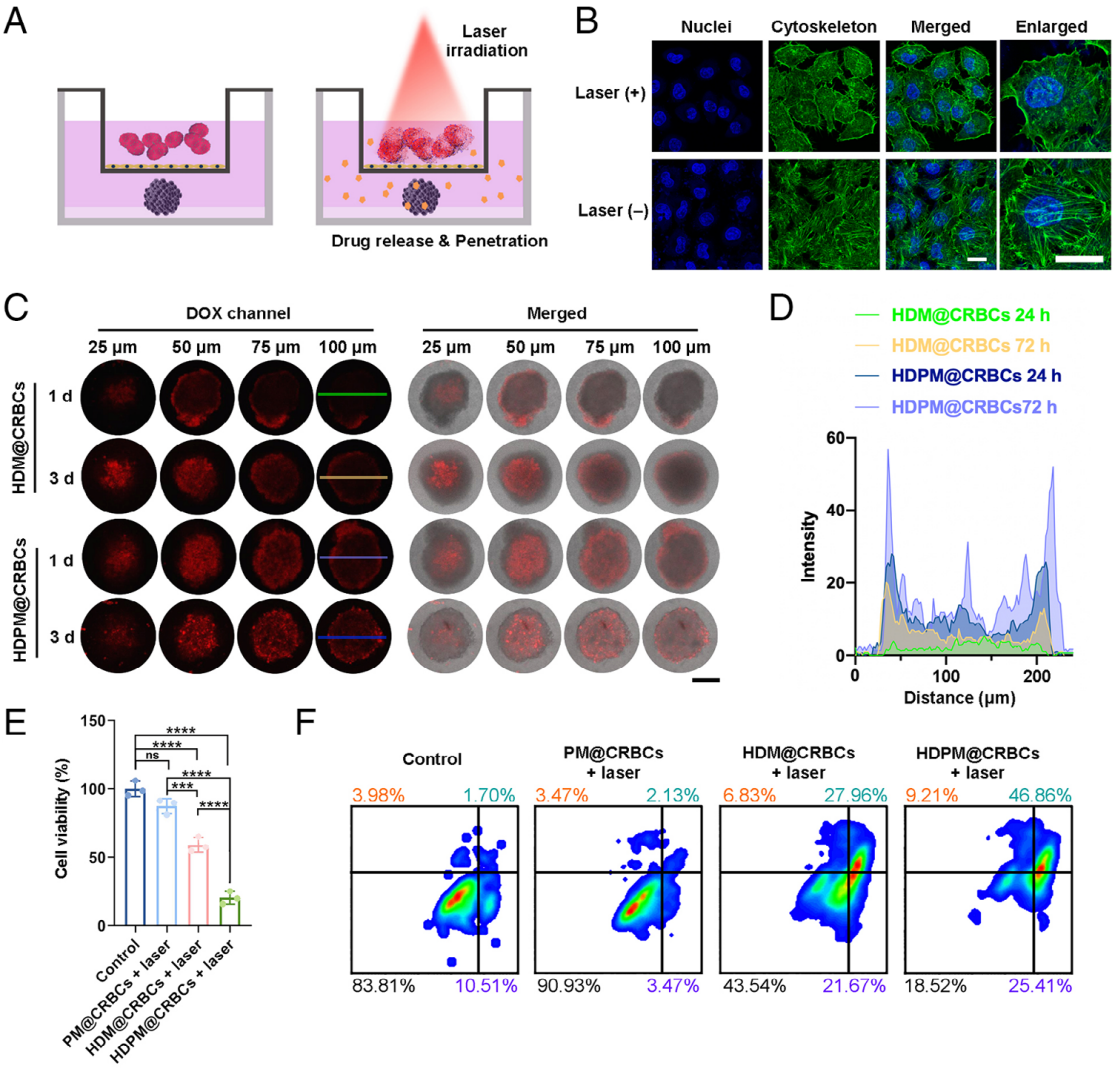
In vitro evaluation of drug penetration and cell killing efficiencies of HDPM@CRBCs. A, Schematic illustration of the transwell assay testing the ability of the released payloads from RBCs to penetrate through the monolayer of HUVEC on the upper chamber and to act on the MTS in the lower chamber. B, Confocal fluorescence images of HDPM@CRBC‐treated HUVEC with or without laser irradiation (808 nm, 300 mW cm^−^^2^, 10 min). Before imaging, the cells were subjected to cytoskeleton and cell nucleus staining. Scale bars: 20 µm. C, Confocal images of MTSs in the lower chambers of transwells at day 1 and day 3 after laser irradiation (808 nm, 300 mW cm^−^^2^, 10 min), the HDM@CRBCs and HDPM@CRBCs were added in the upper chamber of the transwells. Scale bar: 200 µm. D, Corresponding fluorescence intensity profiles of the areas marked by the coloured lines in the confocal images in C. E, Cell viabilities of MTSs in the lower chambers of different groups at day 3 after indicated treatments. F, Flow cytometric results indicating the apoptosis/necrosis levels of MTSs in the lower chambers of transwells at day 3 after different treatments. Statistical data are presented as mean ± standard deviation (*n* = 3) and analyzed by one‐way analysis of variance (ANOVA) with Tukey's post‐hoc test (***p < 0.001, ****p < 0.0001). “ns” stands for non‐significance.

We then investigated the in vitro anticancer efficiency of HDPM@CRBCs using the MTS model. We deduced that the HDPM@CRBCs could be guided to the tumour site under magnetic field and accumulate in tumour vessels. Upon laser irradiation, the HDPM@CRBCs could release their payloads and generate heat to disrupt the vascular wall for the extravasation of the released drugs. To mimic this process in vitro, we established a transwell model where the upper chamber was seeded with a monolayer of human umbilical vein endothelial cells (HUVEC) and the MTS was placed in the lower chamber (Figure 2A). As shown in Figure 2B, after laser irradiation, the photothermal effect of HDPM@CRBCs efficiently disrupted the cytoskeleton of HUVEC, and led to the formation of gaps between adjacent cells, ensuring the passage of the released drugs. Then, we investigated the drug penetration and cell killing efficiencies of HDPM@CRBCs toward the MTSs seeded in the lower chambers. As expected, the “HDPM@CRBCs + laser” group showed deeper drug penetration (Figure 2C,D) and guaranteed a better tumour cell killing efficiency (Figure 2E) compared with the PFD‐free counterpart. Flow cytometric analysis also uncovered that the MTSs in the “HDPM@CRBCs + laser” group experienced a higher level of cell apoptosis or necrosis than those in other groups (Figure 2F). Taken together, the above results suggest that HDPM@CRBCs can disrupt the integrity of the vascular wall under laser irradiation and enable their released drugs to enter solid tumours.

**FIGURE 3:**
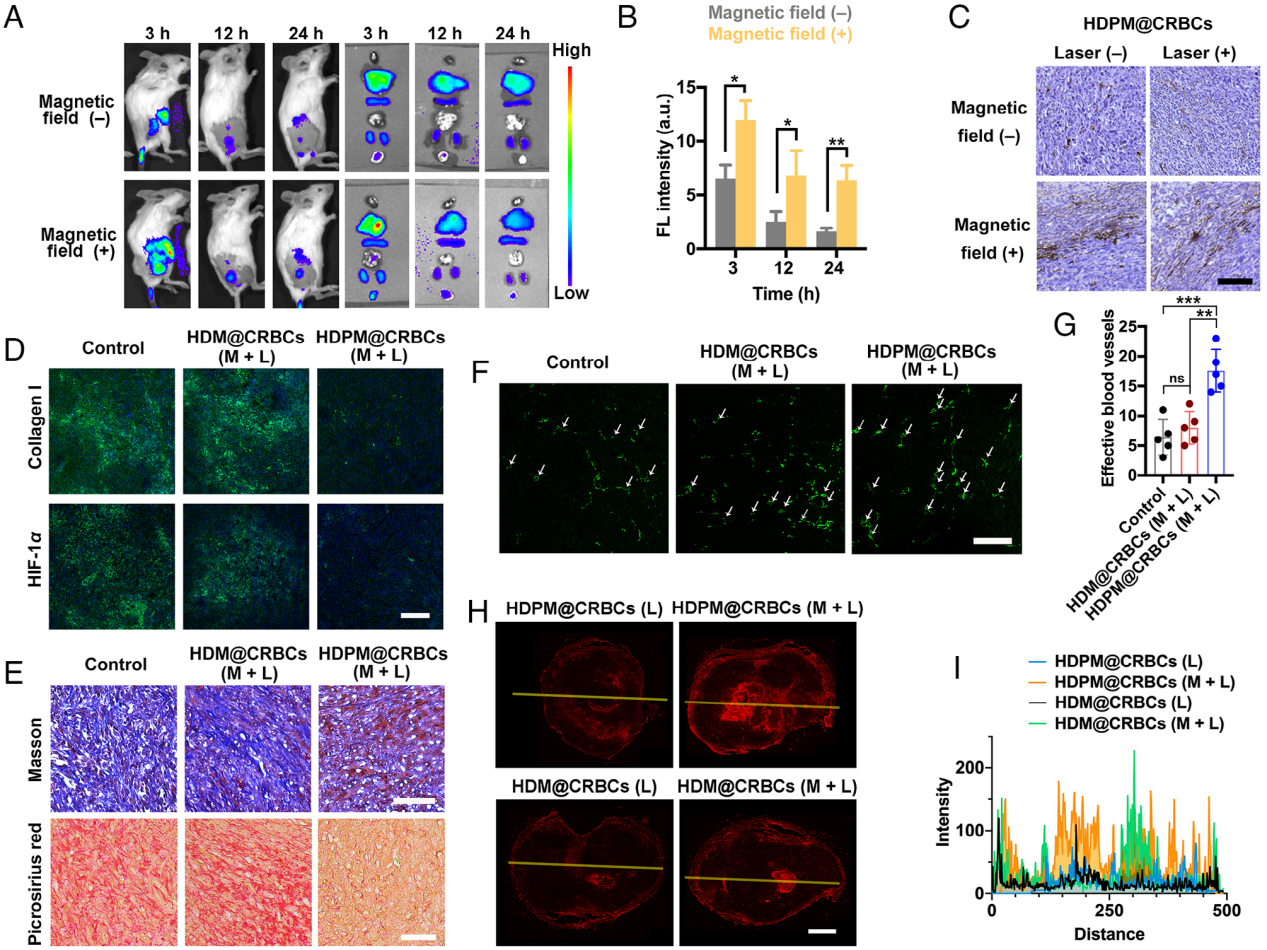
Efficient tumour accumulation and penetration of DOX@HSA delivered by HDPM@CRBCs. A, Time‐dependent in vivo fluorescence images of tumour‐bearing mice that were intravenously injected with HDPM@CRBCs and then treated with or without a tumour‐localized magnetic field. B, Quantitative fluorescence intensities of tumours from the mice receiving the treatments as described in A. Data are presented as mean ± standard deviation (*n* = 3) and analyzed by student's *t*‐test (*p < 0.05, **p < 0.01). C, DAB‐enhanced Prussian blue‐stained tissue slices of tumours collected from the mice 3 days after injection of HDPM@CRBCs. For laser (+) groups, the mouse tumour regions were irradiated by an 808 nm laser for 20 min at 4 h post injection. For magnetic field (+) groups, a magnet was installed on the tumour region of each mouse to provide a localized magnetic field. D, Confocal images revealing the immunofluorescence signals of collagen I and hypoxia‐inducible factor‐1α (HIF‐1α) in the tumours collected from mice 3 days after intravenous injection of PBS (control), HDM@CRBCs, or HDPM@CRBCs. “M + L” stands for magnetic guidance and laser irradiation. Scale bar: 100 µm. E, Masson's trichrome‐ and picrosirius red‐stained tissue slices of the tumours collected from the mice receiving the identical treatments as described in D. Scale bar: 100 µm. F, Immunofluorescence staining results of CD31 molecules in the tumours collected from the mice receiving the identical treatments as described in D. Effective tumour blood vessels are marked by white arrows. Scale bar: 100 µm. G, Average numbers of the effective blood vessels in the tumour slices quantified according to F. Data are presented as mean ± standard deviation (*n* = 5) and analyzed by one‐way ANOVA with Tukey's post‐hoc test (**p < 0.01, ***p < 0.001), “ns” stands for non‐significance. H, Fluorescence images of tumour slices collected from the mice 3 days after injection of HDM@CRBCs or HDPM@CRBCs. M and L stand for magnetic guidance and laser irradiation, respectively. The red fluorescence signals were from DOX in DOX@HSA. Scale bar: 2 mm. I, Corresponding fluorescence intensity profiles of the areas marked by the yellow lines in H.

In our design, the HDPM@CRBCs should be recruited to the tumour site under magnetic guidance so that their loaded drugs can be released in a light‐controlled and site‐specific fashion. To demonstrate this premise, we first evaluated the in vivo tumour accumulation efficiency of HDPM@CRBCs in tumour‐bearing mice by monitoring their fluorescence biodistribution in the presence of an external magnetic field. As shown in Figure 3A, HDPM@CRBCs were substantially recruited at the tumour site with the help of magnetic guidance, while the identically treated mice without magnetic guidance exhibited only a moderate level of drug accumulation at the tumour tissue. Quantitative results further revealed that additional magnetic guidance significantly prolonged the retention time of tumour‐located HDPM@CRBCs (Figure 3B). Besides, diaminobezidin (DAB)‐enhanced Prussian blue staining, which can reflect the presence of Fe_3_O_4_ NPs, also proved the evident tumour accumulation of HDPM@CRBCs (Figure 3C). Therefore, the above results demonstrate the necessity of magnetic guidance for improving the tumour accumulation of HDPM@CRBCs.

It has been reported that the existence of ECM can squeeze tumour vessels and reduce the tumour blood flow, which contributes to tumour hypoxia and hinders the tissue penetration of delivered drugs. We inferred that the PFD released from HDPM@CRBCs upon laser irradiation could inhibit the formation of tumour ECM and therefore help DOX@HSA to more efficiently enter the tumour. To demonstrate the effect of PFD on ECM formation, we evaluated the expression of collagen I, a major component of ECM, by immunofluorescence staining. The results in Figure 3D indicated that the HDPM@CRBCs (M + L) (M: magnetic field, L: laser irradiation) treatment significantly lowered the level of collagen I expression compared with the other groups. This observation was further validated by the Masson's trichrome staining and picrosirius red staining results (Figure 3E), which clearly indicated the loss of collagen fibres in the tumour from the “HDPM@CRBCs (M + L)”‐treated mice. The inhibition of tumour ECM formation was found to improve the abundance of local blood vessels, as evidenced by the increased immunofluorescence signals of CD31 molecules (Figure 3F,G). The normalization of tumour vasculature resulted in enhanced O_2_ supply and the relief of tumour hypoxia (Figure 3D), as evidenced by the reduced expression of HIF‐1α. Importantly, the inhibited ECM formation ensured the concomitantly released DOX@HSA to readily enter the core region of tumour tissues (Figure 3H,I). Collectively, these results reveal the critical role of PFD in loosening tumour ECM and ensuring the deep tissue penetration of DOX@HSA.

**FIGURE 4:**
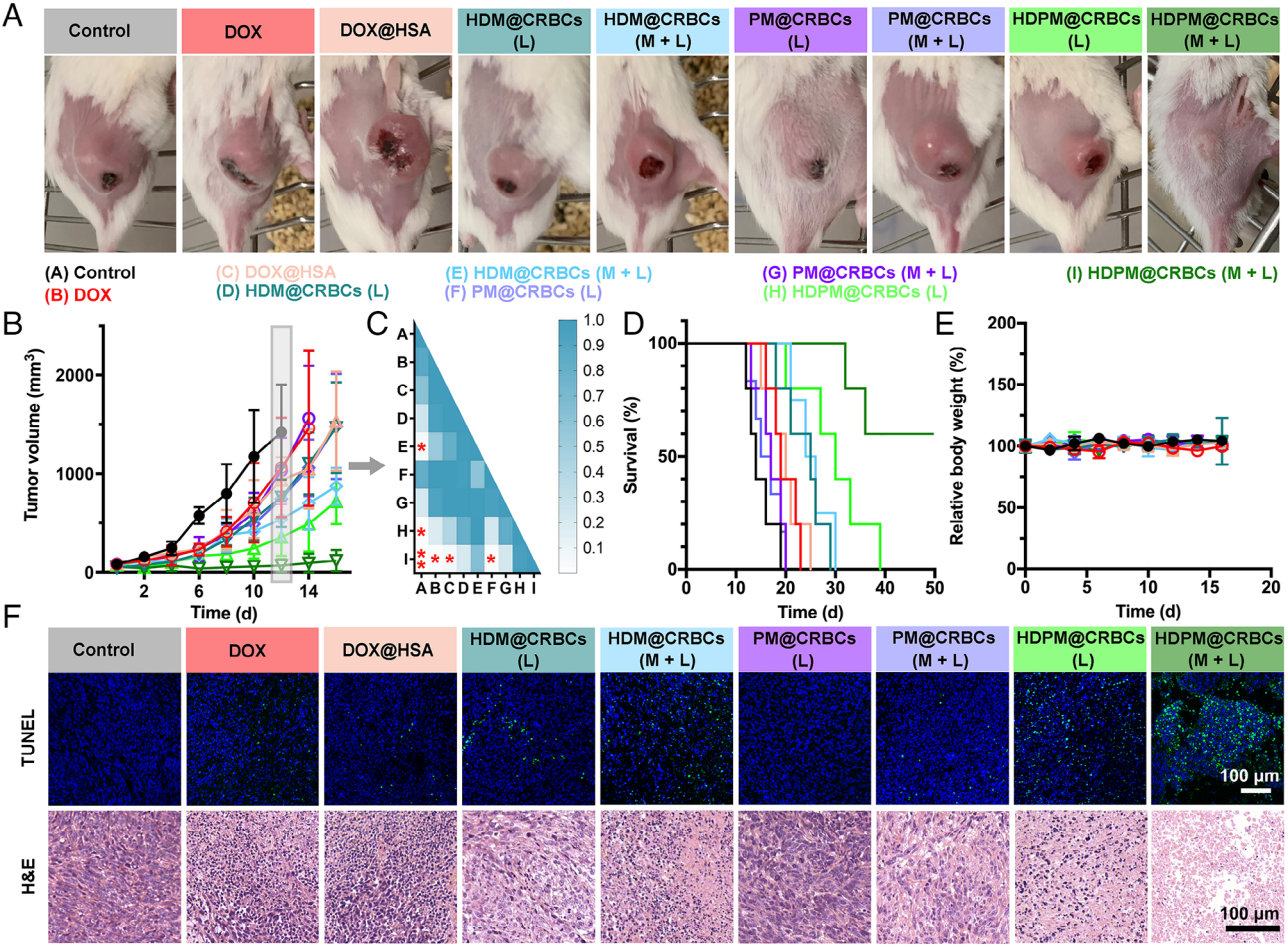
In vivo antitumour evaluation of HDPM@CRBCs. A, Representative photographs and B, tumour growth curves of different groups. C, *p* value heatmap illustrating the statistical significance of the tumour volumes from different groups at day 12, as analyzed by one‐way ANOVA with Tukey's post‐hoc test (*p < 0.05, **p < 0.01, and the non‐significant difference was not labelled by * or **). D, survival rates of mice after various treatments as indicated. E, Relative body weight curves of the mice from indicated groups. F, TUNEL and H&E‐stained tissue slices of tumours collected from the mice which were sacrificed on the 14th day after various treatments as indicated. “M” indicates magnetic field guidance for 4 h at the tumour site, “L” indicates laser irradiation (808 nm, 300 mW cm^−^^2^, 20 min) at 4 h post injection, and “M + L” indicates magnetic field guidance for 4 h after drug injection and subsequent laser irradiation (808 nm, 300 mW cm^−^^2^, 20 min).

Inspired by the efficient tumour accumulation and drug release of HDPM@CRBCs under magnetic guidance and laser control, we then tested the tumour ablation efficacy of HDPM@CRBCs in 4T1 tumour‐bearing mice. As demonstrated above, the introduction of PFD enhanced the drug penetration and relieved tumour hypoxia by inhibiting ECM formation. Thus, we reason that HDPM@CRBCs are promising to achieve an excellent tumour ablation outcome. As expected, the “HDPM@CRBCs (M + L)” group showed a better anticancer outcome than the other groups, as indicated by the slow tumour growth trend and prolonged lifetime of the tumour‐bearing mice (Figure 4A–D), and did not cause significant body weight changes (Figure 4E). Although some control groups, such as “HDPM@CRBCs (L)”, “HDM@CRBCs (L)”, and “HDM@CRBCs (M + L)”, also exhibited a certain degree of inhibitory effect toward tumour growth, these treatments could not realize long‐term inhibition to tumours over the course of our observation, justifying the irreplaceable role of each component in the HDPM@CRBC system. After treatments, all mice were sacrificed and their tumours were processed for pathological analyses, including haematoxylin and eosin (H&E) staining as well as the terminal deoxynucleotidyl transferase‐mediated deoxyuridine triphosphate (dUTP) nick‐end labelling (TUNEL) assay. The results in Figure 4F indicated that the “HDPM@CRBCs (M + L)” treatment efficiently damaged the tumour tissue and caused extensive tumour cell apoptosis with severe DNA fragmentation. In addition, we further proceeded to evaluate the levels of tumour metastasis in mice after different treatments. The H&E and Indian ink staining results revealed the presence of evident metastatic nodules in the lungs of the control mice, while the “HDPM@CRBCs (M + L)” treatment substantially inhibited the metastasis of primary tumours (Figure S8a). Furthermore, the biosafety evaluation results demonstrated that systemic administration of HDPM@CRBCs in mice did not incur apparent adverse effects toward their major organs (Figure S8b) or significantly deviate the values of several haematological and biochemical parameters, including white blood cells (WBC), red blood cells (RBC), haemoglobin (HGB), haematocrit (HCT), platelet (PLT), alanine transaminase (ALT), aspartate amino‐transferase (AST), blood urea nitrogen (BUN), and creatinine (CRE), from their normal levels (Figure S8c). Collectively, the above data demonstrate that HDPM@CRBCs can inhibit both primary and metastatic tumours under the control of dual external fields and possess desirable biosafety profiles.

## 3 CONCLUSION

In this work, we developed an “all‐in‐one” microrobot HDPM@CRBC by conjugating a photothermal agent, cypate, onto the surface of an RBC and encapsulating Fe_3_O_4_ NPs, DOX@HSA, and PFD into its interior cavity. The HDPM@CRBCs originated from native RBCs and inherited their major components and good compatibility. The similar size and shape between HDPM@CRBCs and native RBCs also endowed the system with long circulation time. Under the guidance of a localized magnetic field, the HDPM@CRBCs could be spatiotemporally controlled to accumulate at the tumour site. Subsequent NIR laser irradiation triggered the disintegration of HDPM@CRBCs and guaranteed efficient in situ drug release. The released PFD enhanced the penetration depth of DOX@HSA by inhibiting the formation of ECM, achieving substantial elimination of both primary and metastatic tumours. Moreover, the microrobots are suitable for delivering a wide range of nanodrugs and small‐molecule drugs, including hydrophilic and hydrophobic ones. To summarize, this work displays a hierarchically responsive microrobot capable of meeting the requirement of different steps during tumour‐targeted drug delivery and, more broadly, provides a promising example for the fabrication of intelligent micro/nanorobots.

## AUTHOR CONTRIBUTIONS

Ya‐Xuan Zhu, Hao‐Ran Jia, and Fu‐Gen Wu designed the experiments. Ya‐Xuan Zhu, Hao‐Ran Jia, Yao‐Wen Jiang, Yuxin Guo, Qiu‐Yi Duan, Ke‐Fei Xu, Bai‐Hui Shan, Xiaoyang Liu, and Xiaokai Chen performed the experiments. Ya‐Xuan Zhu, Hao‐Ran Jia, and Fu‐Gen Wu carried out the data analysis and wrote the manuscript. Fu‐Gen Wu supervised the research. All authors proofed and approved the final version of this manuscript for submission.

## CONFLICT OF INTEREST STATEMENT

The authors declare no conflicts of interest.

## Supporting information

Supporting Information
The Supporting Information is available free of charge at
Experimental section and additional figures including grafting quantity, grafting rate, fluorescent intensity, temperature change, haemolysis rate, FSC and SSC signals of M@CRBCs, flow cytometric results, and confocal images before and after magnetic separation, movement trajectories of M@CRBCs, confocal images and quantitative analysis, cell viabilities of MTSs, lung metastasis inhibition, and safety evaluations (PDF)

## Data Availability

All data needed to support the findings of this study are present in the paper and/or in the Supporting Information. Additional data related to this paper are available from the corresponding authors.
