# Supplementary material for: A red blood cell‐derived bionic microrobot capable of hierarchically adapting to five critical stages in systemic drug delivery
Source: Exploration (Beijing). 2023 Dec 10;4(2):20230105. doi: 10.1002/EXP.20230105 (PMC11022606; doi:10.1002/EXP.20230105)
Supplement: Supplementary file 1 — Supporting Information The Supporting Information is available free of charge at Experimental section and additional figures including grafting quantity, grafting rate, fluorescent intensity, temperature change, haemolysis rate, FSC and SSC signals of M@CRBCs, flow cytometric results, and confocal images before and after magnetic separation, movement trajectories of M@CRBCs, confocal images and quantitative analysis, cell viabilities of MTSs, lung metastasis inhibition, and safety evaluations (PDF) [file EXP2-4-20230105-s001.docx]

Supporting Information

A red blood cell-derived bionic microrobot capable of hierarchically adapting to five critical stages in systemic drug delivery

Ya-Xuan Zhu, Hao-Ran Jia,* Yuxin Guo, Qiu-Yi Duan, Ke-Fei Xu, Bai-Hui Shan, Xiaoyang Liu, Xiaokai Chen, Fu-Gen Wu*

**Materials and Methods**

*Materials:* Cypate was synthesized according to a previously reported method.^[1,2]^ *N*-Hydroxysulfosuccinimide sodium salt (sulfo-NHS), 1-ethyl-3-(3-dimethylaminopropyl)-carbodiimide hydrochloride (EDC•HCl), pirfenidone (PFD), and 2-(*N*-morpholino)ethanesulfonic acid (MES) were ordered from Aladdin Chemistry Co., Ltd. (Shanghai, China). Fe_3_O_4_ nanoparticles (NPs) were purchased from Nanjing XFNANO Materials Tech Co., Ltd. (Nanjing, China). Dimethyl sulfoxide (DMSO) was purchased from Shanghai Lingfeng Reagent Chemical Co., Ltd. (Shanghai, China). Cyanine3 *N*-hydroxysuccinimide ester (NHS-Cy3) and cyanine5 *N*-hydroxysuccinimide ester (NHS-Cy5) were purchased from Nanjing Bioorth Biotech Co., Ltd. (Nanjing, China). Hoechst 33342 was bought from Beyotime Institute Biotechnology (Shanghai, China). ActinGreen and cell counting kit-8 (CCK-8) were purchased from KeyGEN BioTECH (Nanjing, China). Doxorubicin hydrochloride (DOX) was purchased from Beijing HVSF United Chemical Materials Co., Ltd. Human serum albumin (HSA) was purchased from Sigma-Aldrich (Shanghai, China). Alsever’s solution was purchased from Beijing Solarbio Science & Technology Co., Ltd. (Beijing, China). Annexin V-fluorescein isothiocyanate (FITC)/propidium iodide (PI) apoptosis detection kit was obtained from US EVERBRIGHT Inc. (Suzhou, China). Anti-CD31 antibody and anti-HIF-1α antibody were obtained from Cell Signaling Technology Inc. (Berverly, MA, USA), and anti-collagen I antibody and FITC-labeled goat anti-rabbit IgG antibody were obtained from Servicebio Technology Co., Ltd. (Wuhan, China). Dialysis membranes (molecular weight cut-off (MWCO): 1 and 8 kDa) were ordered from Spectrumlabs, Inc. (Rancho Dominguez, CA, USA). Deionized water (18.2 MΩ cm) was obtained from a Milli-Q system (Millipore, Billerica, MA, USA).

*Isolation of red blood cells (RBCs):* To obtain RBCs, the whole blood collected from healthy BALB/c mice was centrifuged at 1500 rpm for 5 min to remove the plasma. After washed with cold phosphate-buffered saline (PBS) for 3 times, the RBCs were stored in Alsever’s solution at 4℃ for further use.

*Preparation of cypate-modified RBCs (CRBCs):* To prepare CRBCs, 2 mg cypate was first dissolved in 20 μL of DMSO and then diluted to 1 mg mL^–1^ using water. To activate cypate molecules, the cypate solution was added with sulfo-NHS and EDC•HCl at an cypate:sulfo-NHS:EDC•HCl molar ratio of 1:5:5, and the pH of the obtained mixture was adjusted to 6.0 using MES buffer, followed by reaction at room temperature for 1 h. Next, the activated cypate was harvested by centrifugation (12000 rpm, 10 min) and washed with MES buffer for at least 3 times. The obtained precipitate was dissolved in 20 μL of DMSO and then diluted to 1 mg mL^–1^ (quantified by the ultraviolet−visible (UV−vis) spectroscopy using a spectrophotometer (UV-2600, Shimadzu, Japan)) using PBS for immediate use. After that, 1 mL of RBCs was dispersed in 8.5 mL of PBS and mixed with 0.5 mL of the above activated cypate solution to reach different final cypate concentrations of 10, 20, 30, 50, 75, 100, 150, and 200 μg mL^–1^. The resultant mixtures were kept at 25℃ under shaking for 2 h and centrifuged at 1500 rpm for 5 min to obtain the CRBCs.

*Preparation of drug-loaded CRBCs:* To prepare HDPM@CRBCs, 300 μL of CRBCs was first dispersed in 700 μL of PBS solution. Next, 200 μL of the above-obtained suspension was mixed with 1 mL of PBS solution containing 200 μg Fe_3_O_4_ NPs, 2 mg PFD, and 5 mg DOX@HSA. Then, the mixture was dialyzed against 100 mL of the hypotonic buffer (10 mM NaHCO_3_, 10 mM NaH_2_PO_4_, 20 mM glucose, 2 mM ATP, and 3 mM reduced GSH) at 4℃ for 30 min. Afterwards, the mixture was withdrawn from the dialysis bag and added with the hypertonic solution (100 mM sodium pyruvate, 100 mM inosine, 10 mM glucose, 4 mM MgCl_2_, 190 mM NaCl, 1666 mM KCl, 33 mM NaH_2_PO_4_, and 20 mM ATP) at a volume ratio of hypertonic solution:mixture = 1:9. After incubation at 37℃ for 30 min, the mixture was centrifuged at 3500 rpm for 5 min, and the collected HDPM@CRBCs were washed with cold PBS for 3 times to remove the unloaded drugs and nanoparticles. The HDM@CRBCs and PM@CRBCs were also prepared via the similar methods. The amounts of DOX and PFD in HDPM@CRBCs, HDM@CRBCs, and PM@CRBCs were quantified by a fluorescence spectrophotometer (Duetta, HORIBA, Japan) and an Agilent 1260 Infinity II high-performance liquid chromatography (HPLC) system, respectively.

*Characterization of CRBCs:* To confirm the successful conjugation of cypate, CRBCs were observed by an inverted confocal laser scanning microscope (TCS SP8, Leica, Germany) with a 63× oil immersion objective. To measure the grafting efficiency of cypate, CRBCs at a volume of 100 μL were dispersed in water (1 mL) for 30 min. Then, the CRBC membranes were obtained by centrifugation (8000 rpm, 10 min), washed with water for 3 times, and suspended in a 0.1% Triton-X 100 solution. The concentration of cypate was quantified by UV−vis spectroscopy. The fluorescence intensities of CRBCs were quantified using a flow cytometer (NovoCyte 2070R, ACEA Biosciences, Inc., USA). The morphology of HDPM@CRBCs was investigated using a field emission scanning electron microscope (Ultra Plus, Zeiss, Germany).

*Magnetic separation efficiency:* To investigate the magnetic separation efficiency of CRBCs loaded with different amounts of Fe_3_O_4_ NPs, we first labeled the RBCs with Cy3 and Cy5 to obtain Cy3-labeled RBCs (Cy3-RBCs) and Cy5-labeled RBCs (Cy5-RBCs), respectively. Then, the Cy5-labeled RBCs were loaded with different concentrations of Fe_3_O_4_ NPs (Fe_3_O_4_@Cy5-RBCs) according to the procedures described above. Then, the Cy3-RBCs and Fe_3_O_4_@Cy5-RBCs were mixed together and tested by flow cytometry and confocal laser scanning microscopy before and after magnetic separation. To demonstrate that the HPDM@CRBCs can be efficiently separated from blood by magnetic field, the HDPM@CRBCs (APC-Cy7-positive) were first mixed with natural RBCs (APC-Cy7-negative) at a ratio of 7:3, and subjected to a magnetic field. After magnetic separation, a part of cells were removed from the mixture, and the remaining mixture was collected. The ratios of HDPM@CRBCs and natural RBCs were quantified by flow cytometric analysis before and after magnetic separation.

*Movement trajectory of M@CRBCs:* To demonstrate the magnetic field-guided movement of M@CRBCs, the M@CRBCs were first dropped onto a glass slide. Then, a magnet was placed 0.3 or 0.8 cm away from the droplet in different directions, and the locations of particles of interest were recorded by a microscope every second.

*Photothermal performance evaluation:* RBCs (with a volume of 10 μL) reacted with different concentrations of cypate were separately suspended in 200 μL of PBS solution, and the obtained suspensions were irradiated by a 808 nm laser (300 mW cm^–2^). The temperature values were recorded by a thermal imaging camera (FLIR T540, Teledyne, USA).

*Hemolysis assays:* To evaluate the light-triggered hemolysis efficiency, RBCs (10 μL) reacted with different concentrations of cypate were separately dispersed in 200 μL of PBS. Then the obtained suspensions were irradiated by an 808 nm laser (300 mW cm^–2^, 10 min). The RBCs without cypate modification suspended in PBS or H_2_O were set as negative control or positive control. Next, the suspensions were centrifuged at 1500 rpm for 5 min and their supernatants were collected in a 96-well plate. The absorbance of these samples at 570 nm was measured using a microplate photometer (Multiskan FC, Thermo Scientific, USA).

*Drug release measurements:* To investigate the laser-triggered drug release of HDPM@CRBCs, HDPM@CRBCs were first suspended in PBS and kept shaking under 37℃ for 24 h. At different incubation time points (0 min, 5 min, 15 min, 30 min, 1 h, 2 h, 6 h, 12 h, 18 h, and 24 h), 100 μL of the above suspension was withdrawn and centrifuged at 3500 rpm for 5 min, and the amount of DOX in the supernatant was quantified using a spectrofluorophotometer (RF-5301PC, Shimadzu, Japan). Similarly, the PFD release profile was also measured accordingly, and the amount of PFD was quantified by HPLC.

*In vitro therapeutic efficacy evaluated on multicellular tumor sphere (MTS):* To construct the multicellular tumor sphere, 24-well plates were first coated with Dulbecco’s modified Eagle’s medium (DMEM) supplemented with 1.5% agarose and then seeded with 4T1 cells (2.5 × 10^3^ cell/well). After 7 d incubation, the MTSs were obtained.

To evaluate the extracellular matrix (ECM) inhibition effect of PFD, the MTSs were treated with different concentrations of PFD in DMEM for different time periods, and then stained with an ROS-ID Hypoxia/Oxidative Stress Detection Kit (Enzo Life Sciences, USA) to indicate the hypoxia level of MTSs. To verify the penetration depth of DOX@HSA enhanced by PFD, MTSs were incubated with a fixed concentration of DOX@HSA (DOX: 5 μg mL^–1^) and various concentrations of PFD (0, 25, 50, 100, 200, or 400 μg mL^–1^). The DOX fluorescence distribution in MTSs was observed via confocal imaging at different time points after treatment. The cell viabilities of MTSs were investigated according to the procedures described below. Further, the HDM@CRBCs and HDPM@CRBCs were irradiated with a 808 nm laser (300 mW cm^–2^, 10 min) and then centrifuged at 3500 rpm for 5 min. The supernatants were added to the MTSs and the MTSs were observed via confocal imaging at different time points.

To perform transwell assays, an MTS was placed in the lower chamber, and the upper chamber of each transwell (24-well insert; pore size, 400 nm; Corning, USA) was seeded with 5 × 10^4^ human umbilical vein endothelial cells (HUVEC) and kept culture for 7 d to build a compact monolayer of HUVEC. Next, the culture medium in the upper chamber was replaced with fresh culture medium containing HDPM@CRBCs with different DOX concentrations. To conduct laser irradiation, the upper chambers were placed under an 808 nm laser (300 mW cm^–2^) for 10 min. After another 24 h incubation, the MTSs in the lower chambers were collected for further evaluations.

To evaluate the cell viabilities and apoptosis, the MTSs after different treatments were treated with 0.125% trypsin and 0.125% collagenase at 37℃ for 10 min to obtain monodispersed cells. For cell viability evaluation, the monodispersed cells were centrifuged and collected. The cells were then resuspended by DMEM and transferred into a 96-well plate, and the cell viabilities were then evaluated by CCK-8 assay. The absorbance of these samples at 450 nm was measured using the microplate photometer. For apoptosis/necrosis analysis, the monodispersed cells were washed with PBS for 3 times and stained using an annexin V-FITC/propidium iodide (PI) apoptosis kit before being detected by flow cytometry.

*Laser-induced destruction to the HUVEC monolayer:* To investigate the damage of HUVEC by HDPM@CRBCs during laser irradiation, we first cultured HUVEC until the cell confluence reached approximately 100%. Then, the HUVEC were incubated with HDPM@CRBCs (DOX: 5 μg mL^–1^) and irradiated by an 808 nm laser (300 mW cm^–2^) for 10 min. After another 2 h of incubation, the cells were fixed by 4% paraformaldehyde and permeabilized in 0.1% Triton X-100 for 15 min. The cells were then blocked by 3% bovine serum albumin (BSA) at room temperature for 30 min and stained with ActinGreen and Hoechst 33342 (10 μg mL^–1^) for 20 min, followed by PBS washing for 3 times. The stained cells were imaged under the confocal microscope.

*Animal models:* Female BALB/c mice aged 4 weeks were ordered from the Comparative Medicine Center of Yangzhou University (Jiangsu, China). All the animal experiments followed the guidelines of the Animal Care and Ethics Committee of Shanghai Tenth People’s Hospital, Tongji University School of Medicine. The assigned approval/accreditation number: SHDSYY-2022-T0074. To establish tumor-bearing mouse models, 2 × 10^6^ 4T1 murine breast cancer cells suspended in 50 μL of PBS were subcutaneously injected into the back of each mouse. When the volume of tumors reached about 50 mm^3^, the mice were used for the following experiments.

*In vivo and ex vivo fluorescence imaging:* For *in vivo* fluorescence imaging, the tumor-bearing mice were intravenously injected with HDPM@CRBC suspensions (DOX: 1 mg kg^–1^, PFD: 5 mg kg^–1^). Under deep anesthesia by continuous inhalation of a mixture of oxygen with isoflurane (5%), the treated mice were imaged by a PerkinElmer animal imaging system (IVIS Spectrum) at different time points postinjection. For the “magnetic field (+)” group, a magnet was stuck onto the tumor surface before injection. For *ex vivo* fluorescence imaging, the treated mice were sacrificed at 4, 12, or 24 h postinjection and their major organs (hearts, livers, spleens, lungs, and kidneys) and tumor tissues were excised and imaged. PerkinElmer Image Analysis Software was used to quantify the fluorescence signals.

*Immunofluorescence staining and diaminobezidin (DAB)-enhanced prussian blue staining:* Initially, 4T1 tumor-bearing mice were randomly divided into 9 groups and subjected to the following treatments: (1) intravenous (i.v.) injection of PBS, (2) i.v. injection of DOX, (3) i.v. injection of DOX@HSA, (4) i.v. injection of HDM@CRBCs plus laser irradiation, (5) i.v. injection of HDM@CRBCs plus laser irradiation and magnetic field, (6) i.v. injection of PM@CRBCs plus laser irradiation, (7) i.v. injection of PM@CRBCs plus laser irradiation and magnetic field, (8) i.v. injection of HDPM@CRBCs plus laser irradiation, and (9) i.v. injection of HDPM@CRBCs plus laser irradiation and magnetic field. The injection doses of DOX and PFD in these groups were set as 1 mg kg^–1^ and 5 mg kg^–1^, respectively. Laser irradiation was carried out at 4 h postinjection using an 808 nm laser (300 mW cm^–2^, 20 min). Next, the treated mice were sacrificed on the 3rd day and their tumors were excised, followed by frozen section preparation. The obtained tumor slices were permeabilized in 0.1% Triton X-100 for 15 min and then blocked by 3% BSA at room temperature for 3 h. For CD31, collagen I, and hypoxia-inducible factor 1α (HIF-α) staining, the slices were first incubated with the solutions (1/200) of primary antibodies (anti-CD31 antibody, anti-collagen I antibody, or anti-HIF-α antibody) overnight at 4℃. After PBS washing for 3 times, the slices were further incubated with FITC-labeled goat anti-rabbit IgG antibody solutions for 3 h at room temperature. Before confocal imaging, the tumor slices were stained with Hoechst 33342 (10 µg mL^–1^) for 10 min and washed with PBS for 3 times. The slices were also subjected to DAB-enhanced prussian blue staining to indicate the distribution of Fe_3_O_4_ NPs in tumor tissues.

*In vivo antitumor evaluations:* To evaluate the *in viv*o therapeutic efficacy of HDPM@CRBCs, the 4T1 tumor-bearing mice were randomly divided into 10 groups for different treatments: (1) i.v. injection of PBS, (2) i.v. injection of DOX, (3) i.v. injection of DOX@HSA, (4) i.v. injection of HDM@CRBCs plus laser irradiation, (5) i.v. injection of HDM@CRBCs plus laser irradiation and magnetic field, (6) i.v. injection of PM@CRBCs plus laser irradiation, (7) i.v. injection of PM@CRBCs plus laser irradiation and magnetic field, (8) i.v. injection of HDPM@CRBCs plus laser irradiation, and (9) i.v. injection of HDPM@CRBCs plus laser irradiation and magnetic field. The injection doses of DOX and PFD in these groups were set as 1 mg kg^–1^ and 5 mg kg^–1^, respectively. Laser irradiation was carried out at 4 h postinjection using a 808 nm laser (300 mW cm^–2^, 20 min). Tumor volumes and weights of the mice were monitored every 2 days. The tumor volume was calculated as width^2^ × length/2.

*Hematoxylin and eosin (H&E) and terminal deoxynucleotidyl transferase-mediated deoxyuridine triphosphate (dUTP) nick-end labeling (TUNEL) staining:* For the H&E staining of tumor slices, the mice after various treatments were sacrificed on the 14th day and their tumors were excised, followed by fixation in 4% paraformaldehyde solutions. After being embedded in paraffin and sectioned, these samples were stained with H&E following standard protocols. To evaluate the systemic toxicity of HDPM@CRBCs, the healthy BALB/c mice (without tumor inoculation) were injected with 200 μL of PBS or HDPM@CRBC suspension (DOX: 1 mg kg^–1^, PFD: 5 mg kg^–1^). Then, these mice were sacrificed on the 14 day, and their major organs (hearts, livers, spleens, lungs, and kidneys) were excised, followed by H&E staining as described above.

For the TUNEL staining of tumor slices, the treated mice in different groups were sacrificed on the 14th day and their tumors were excised, followed by frozen section preparation. Then, the tumor slices were stained using a TUNEL apoptosis in situ detection kit (KeyGEN BioTECH, Nanjing, China). Before confocal imaging, the slices were stained with Hoechst 33342 (10 µg mL^–1^) for 10 min and washed with PBS for 3 times.

*Evaluation of lung metastatic nodules:* 4T1 tumor-bearing BALB/c mice in different groups were sacrificed at day 14 and their lungs were excised and injected with 15% India ink solutions via the tracheae. After that, the lungs were transferred into Fekete’s solutions (containing 40 mL of glacial acetic acid, 80 mL of formalin, 580 mL of ethanol, and 200 mL of water) for a few minutes to remove the ink adsorbed on nodules. The H&E staining assay was also carried out to evaluate the lung metastatic nodules.

*Hemanalysis and biochemical analysis:* Blood samples were collected from HDPM@CRBC-treated mice (without tumor inoculation) at 21 day postinjection. The blood cells were analyzed by an automatic hematology analyzer (HBVET-1, Sinnowa, China). The biochemical analyses were performed on an automated biochemical analyzer (Chemifastar V, Sinnowa, China). The PBS-treated mice were set as a negative control group.

*Statistical analysis:* Most of the statistical data were presented as the mean ± standard deviation (SD) from at least three independent experiments. Student’s *t*-test was performed to analyze the difference between two groups, and one-way analysis of variance (ANOVA) with Tukey’s post-hoc test was conducted for multiple comparisons, in which a *p* value of less than 0.05 was considered as significant difference.


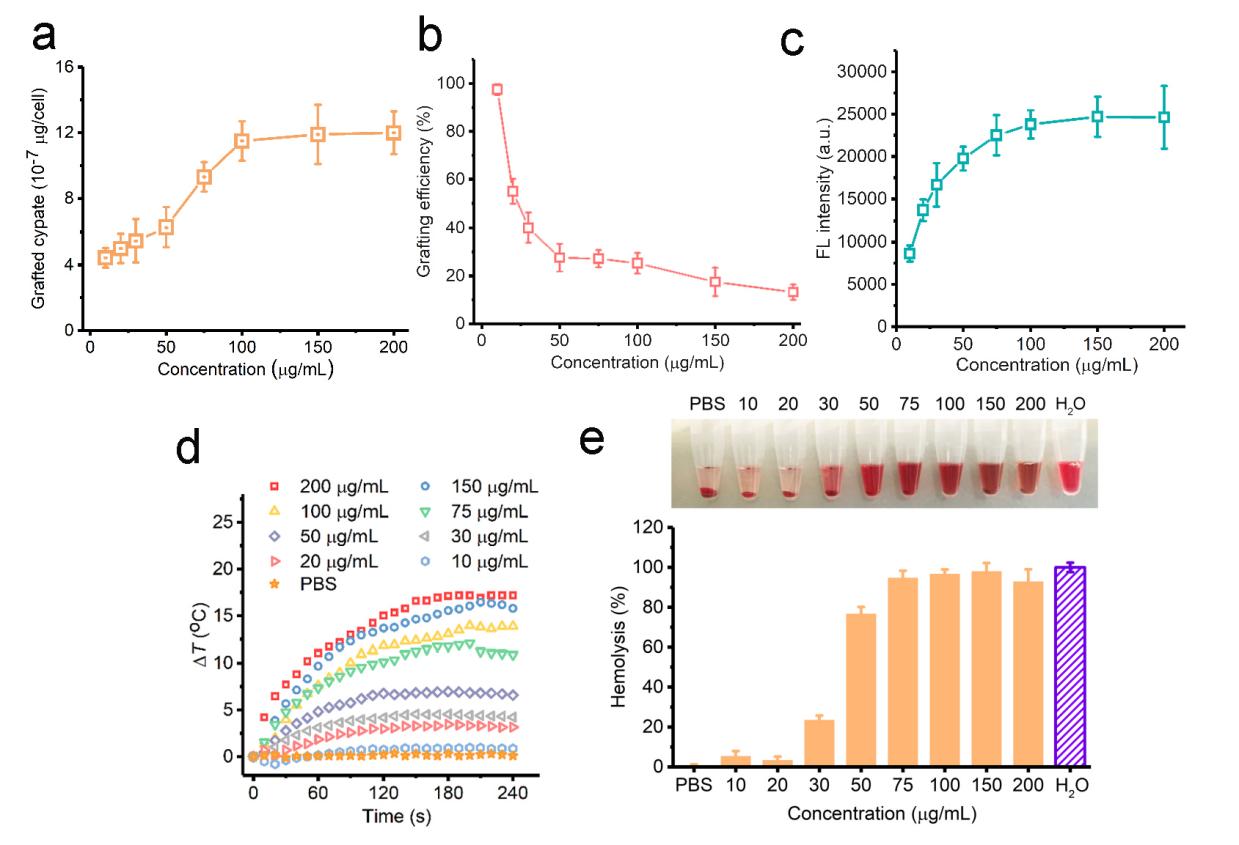


**Figure S1.** (a) Amounts of cypate grafted on a single RBC at different feeding concentrations of cypate. (b) Grafting efficiencies of cypate at different feeding concentrations. (c) Fluorescence (FL) intensities of RBCs measured by flow cytometry after reaction with different concentrations of cypate. (d) Temperature changes of the suspensions of RBCs reacted with different concentrations of cypate during 808 nm laser irradiation (300 mW cm^–2^). (e) Hemolysis rates of RBCs reacted with different concentrations of cypate and irradiated by an 808 nm laser (300 mW cm^–2^, 10 min).


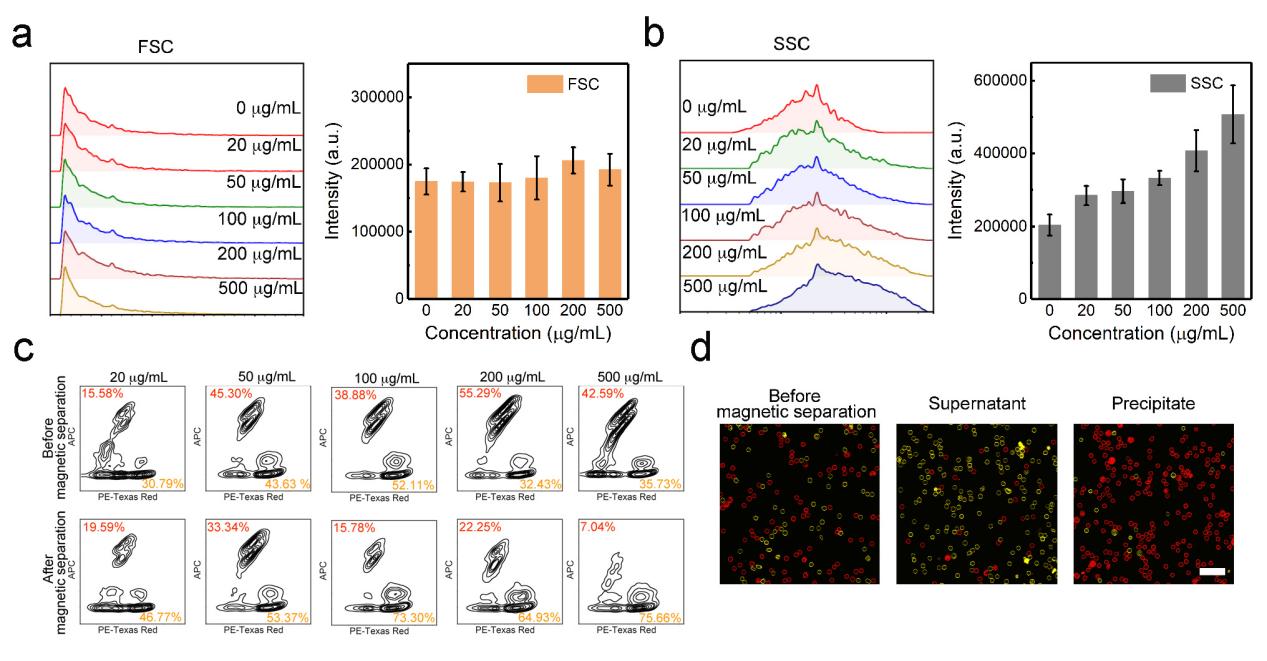


**Figure S2.** (a) Forward scatter (FSC) and (b) side scatter (SSC) signals of RBCs fed with different concentrations of Fe_3_O_4_ NPs and the related quantified results measured by flow cytometry. (c) Flow cytometric results of RBCs fed with different concentrations of Fe_3_O_4_ NPs before and after magnetic separation. The RBCs were labeled with Cy3 (PE-Texas Red channel) and the M@RBCs were labeled with Cy5 (APC channel), and the percentages of Cy3-labeled RBCs and Cy5-labeled M@RBCs were indicated in yellow and red, respectively. (d) Confocal images of RBCs fed with 200 μg mL^–1^ Fe_3_O_4_ NPs. The RBCs were labeled with Cy3 and the M@RBCs were labeled with Cy5.


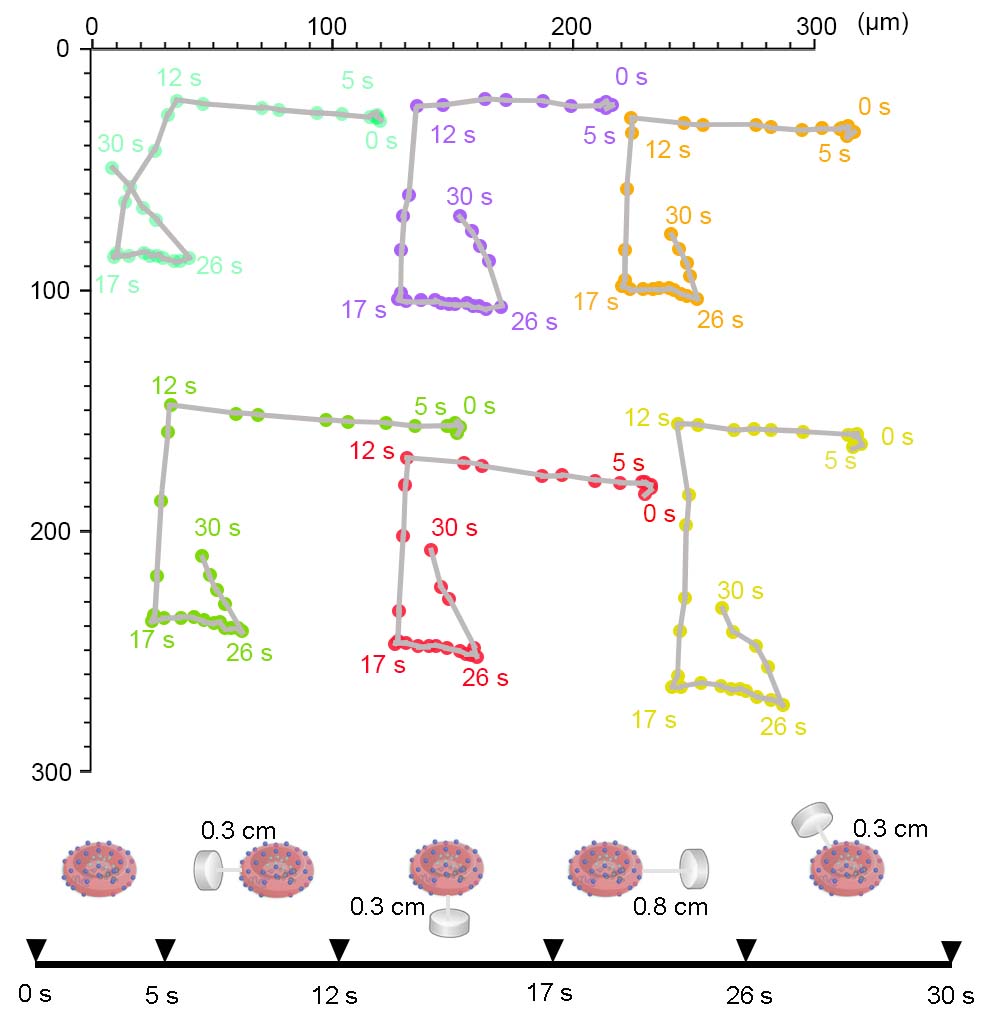


**Figure S3.** Movement trajectories of M@CRBC microrobots under a magnetic field in different directions. The locations of representative particles were recorded by a microscope every second, and the relative positions of the magnet and M@CRBCs were illustrated at the bottom of the figure.


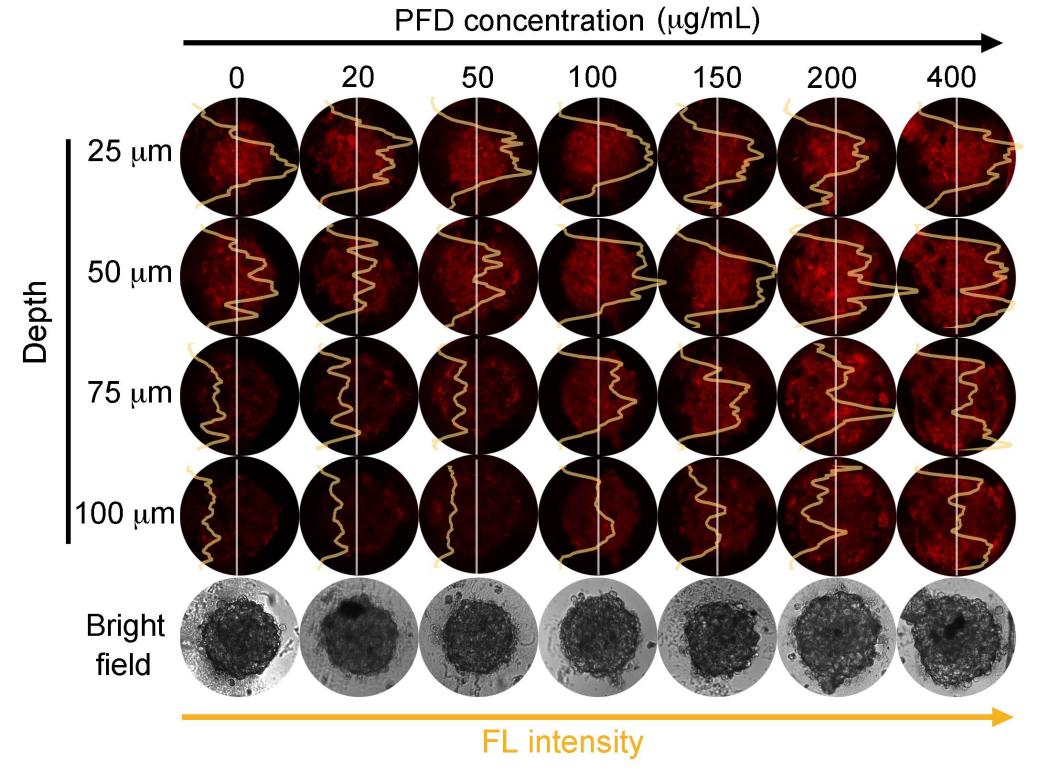


**Figure S4.** Confocal images and quantitative analysis results of the fluorescence intensities along the marked white lines of the MTSs treated with DOX@HSA (DOX: 5µg mL^–1^) and different concentrations of PFD for 1 d.


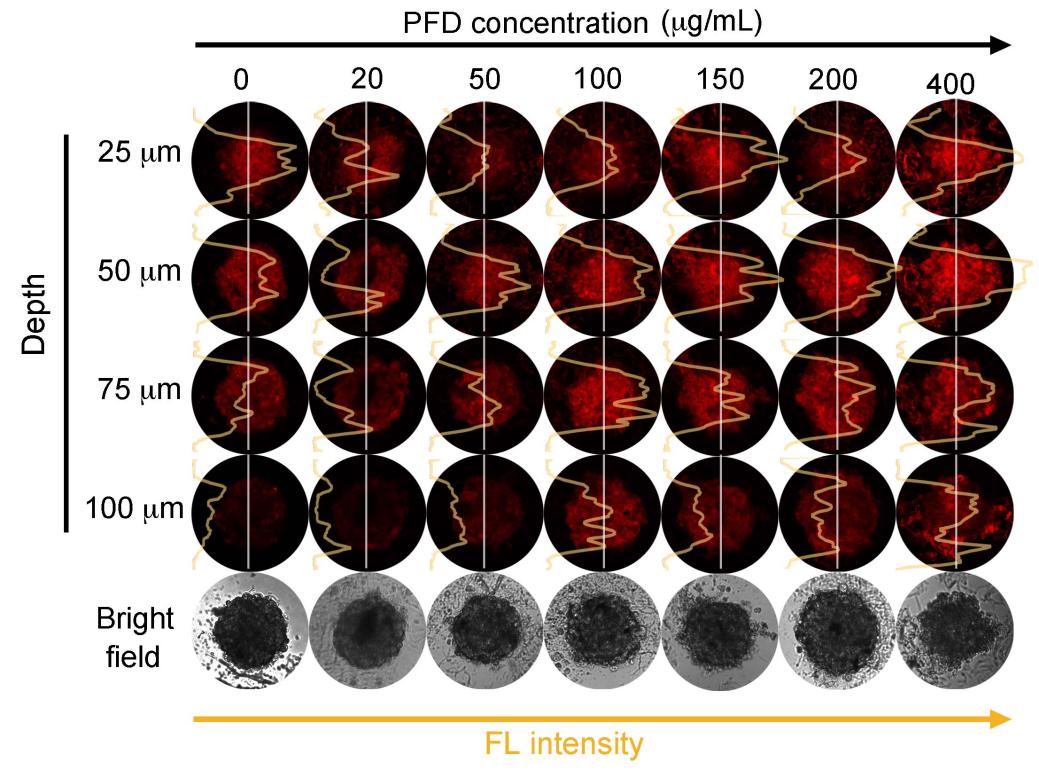


**Figure S5.** Confocal images and quantitative analysis results of the fluorescence intensities along the marked white lines of the MTSs treated with DOX@HSA (DOX: 5 µg mL^–1^) and different concentrations of PFD for 3 d.


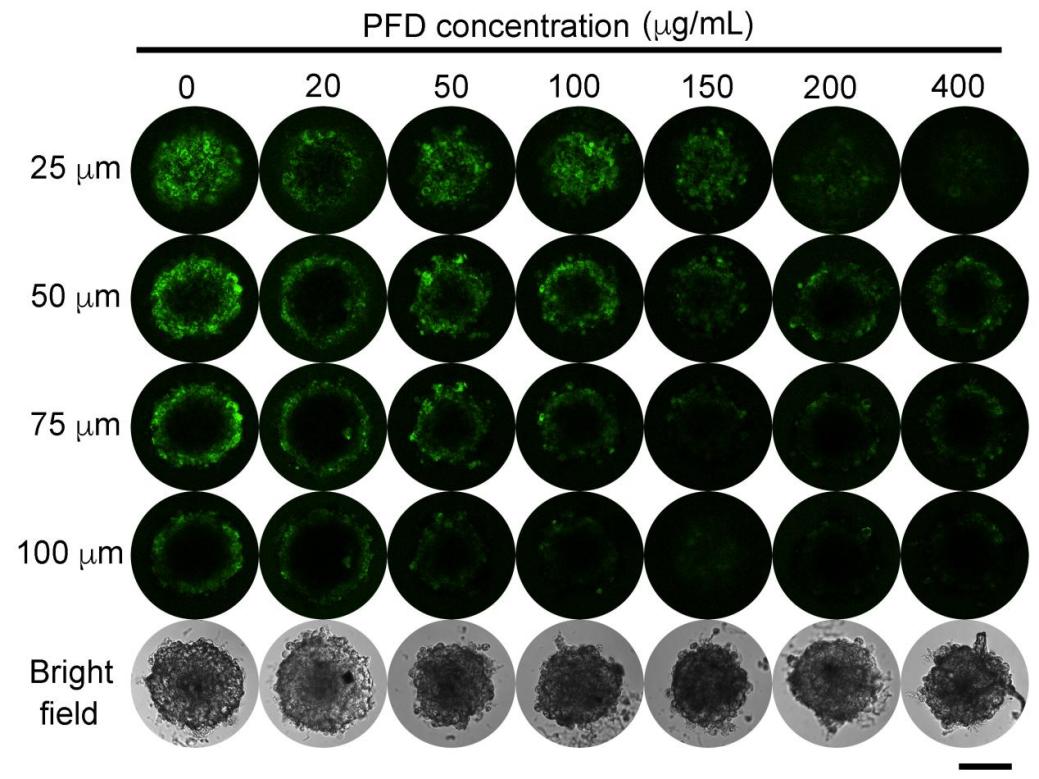


**Figure S6.** Confocal images of the MTSs treated with different concentrations of PFD for 3 d and stained with ROS ID.


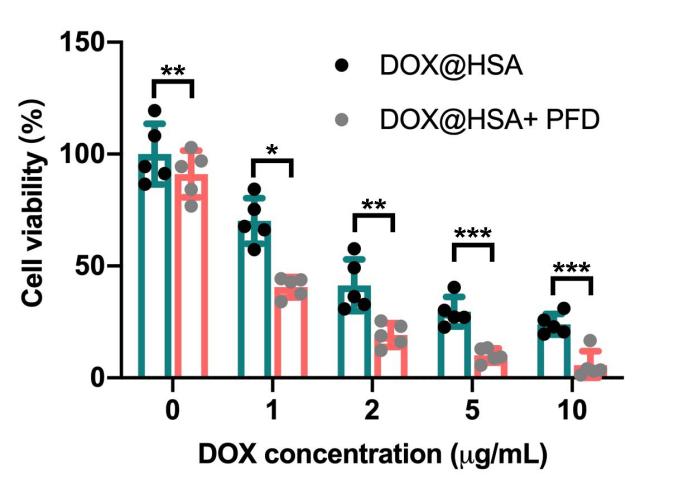


**Figure S7.** Cell viabilities of the MTSs treated with different concentrations of DOX@HSA and PFD (200 μg mL^–1^). The data were obtained via CCK-8 assay. Data are presented as mean ± standard deviation (*n* = 5) and analyzed by student’s *t*-test (**p* < 0.05, ***p* < 0.01, ****p* < 0.001).


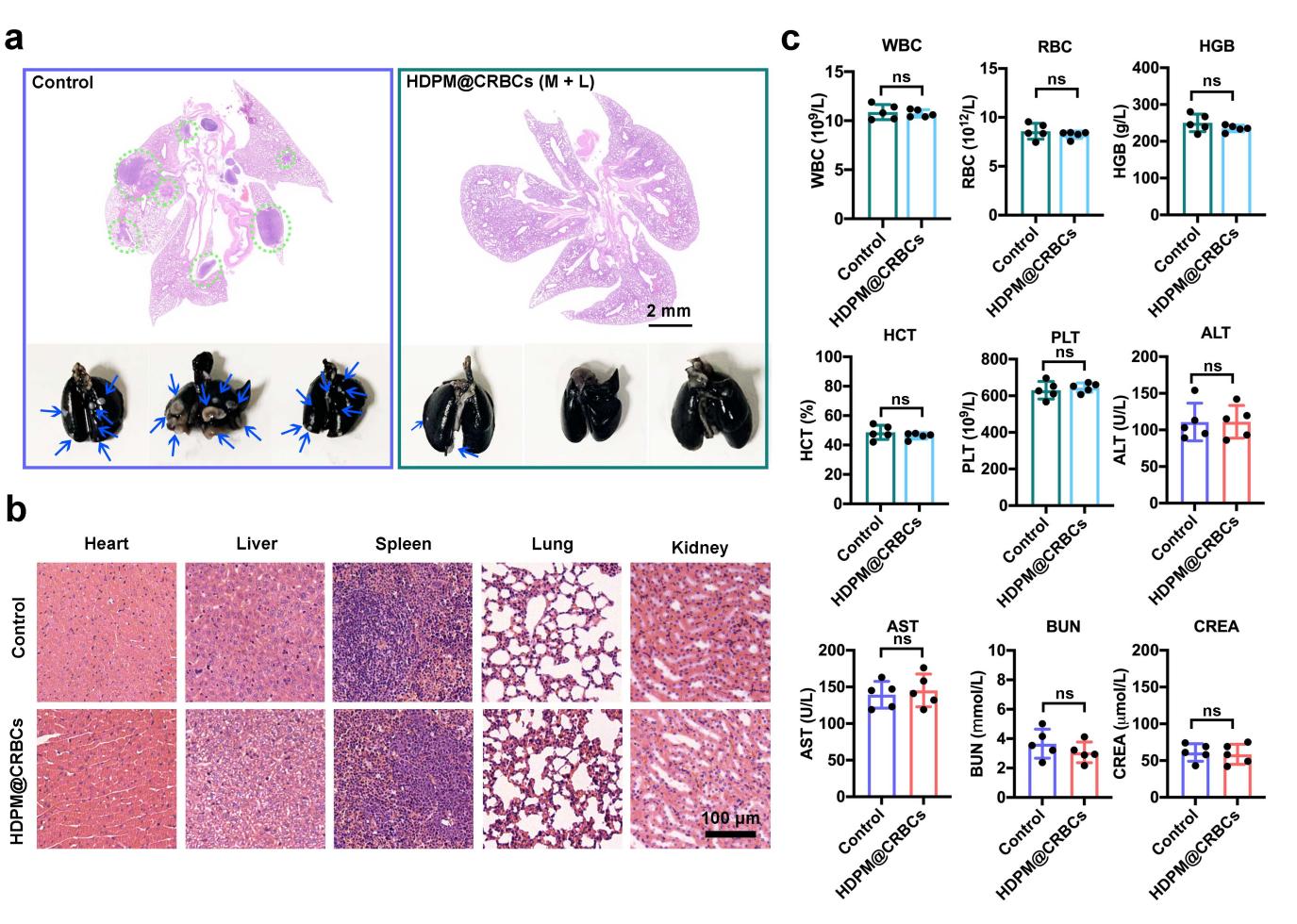


**Figure S8.** Metastasis inhibition and biosafety assessments of HDPM@CRBCs. (a) H&E-stained lung slices and Indian ink-perfused lung tissues of mice from the control and HDPM@CRBCs (M+L) groups. (b) H&E-stained tissue slices of major organs from untreated (control) and HDPM@CRBC-injected healthy mice, which were sacrificed at day 21. (c) Results of hemanalysis and biochemical analysis of untreated (control) and HDPM@CRBC-injected healthy BALB/c mice, whose blood samples were collected and analyzed at day 21. Statistical data are presented as mean ± standard deviation (*n* = 5) and analyzed by student’s *t*-test, “ns” stands for non-significance.

**References**

1. Y. P. Ye, S. Bloch, S. Achilefu, *J*. *Am*. *Chem*. *Soc*. **2004**, *126*, 7740.
2. H. R. Jia, Y. X. Zhu, X. Liu, G. Y. Pan, G. Gao, W. Sun, X. Zhang, Y. W. Jiang, F. G. Wu, *ACS Nano* **2019**, *13*, 11781.
